# Ammonium Ion Exchanged Zeolite for Laser Desorption/Ionization Mass Spectrometry of Phosphorylated Peptides

**DOI:** 10.1155/2015/513761

**Published:** 2015-09-13

**Authors:** Mengrui Yang, Tatsuya Fujino

**Affiliations:** ^1^Institute of Quality Standard and Testing Technology for Agro-Products, Chinese Academy of Agricultural Sciences, Beijing 100081, China; ^2^Department of Chemistry, Graduate School of Science and Engineering, Tokyo Metropolitan University, 1-1 Minami-Osawa, Hachioji, Tokyo 192-0379, Japan

## Abstract

*α*-Cyano-4-hydroxycinnamic acid (CHCA), an organic matrix molecule for matrix-assisted laser desorption/ionization mass spectrometry, was adsorbed to NH_4_^+^-type zeolite surface, and this new matrix was used for the detection of low-molecular-weight compounds. It was found that this matrix could simplify the mass spectrum in the low-molecular-weight region and prevent interference from fragments and alkali metal ion adducted species. CHCA adsorbed to NH_4_^+^-type ZSM5 zeolite (CHCA/NH_4_ZSM5) was used to measure atropine and aconitine, two toxic alkaloids in plants. In addition, CHCA/NH_4_ZSM5 enabled us to detect phosphorylated peptides; peaks of the protonated peptides had higher intensities than the peaks observed using CHCA only.

## 1. Introduction

Matrix-assisted laser desorption/ionization mass spectrometry (MALDI MS) is a soft ionization technique that does not decompose analyte molecules during the ionization process. MALDI MS is widely used to study nonvolatile compounds with high molecular weights, such as peptides and proteins [1]. However, it is seldom applied to the analysis of low-molecular-weight compounds because conventional matrix molecules, such as *α*-cyano-4-hydroxycinnamic acid (CHCA), sinapinic acid (SA), 2,5-dihydroxybenzoic acid (DHB), and 2,4,6-trihydroxyacetophenone (THAP), produce many matrix-related peaks in the low mass region, and those peaks sometimes hamper analyte identification. In order to overcome this drawback, several attempts have been made, such as the use of inorganic materials [2], surfactants [3, 4], nanomaterials [5], and porous materials [6].

Zeolites, which are widely used as catalysts and sorbents, are porous aluminosilicates that consist of a three-dimensional framework of SiO_4_ and AlO_4_ tetrahedrons linked by oxygen bridges. Because of the isomorphous replacement of Si^4+^ by Al^3+^ in the crystal structure, negative charges on the Si–O–Al bridging sites are balanced by cations (H^+^, Na^+^, and K^+^), which are exchangeable with other cations [7, 8]. It is known that hydroxyl groups having strong Brönsted acidity exist in H^+^-type zeolite, and they are responsible for various catalytic reactions on the zeolite surface. In addition, the SiO_2_/Al_2_O_3_ ratio is one of the important factors affecting the catalytic activity. Owing to these properties, zeolites were recently found to be applicable to mass spectrometric analysis; enhancement of the peak intensity of protonated analytes and suppression of the peaks of alkali metal ion adducted species were observed [9, 10]. It was also found that Na^+^-, K^+^-, and Li^+^-type zeolites were applicable to the ionization of low-molecular-weight compounds, which had been difficult to ionize by conventional MALDI MS [11–13].

**Scheme 1:**
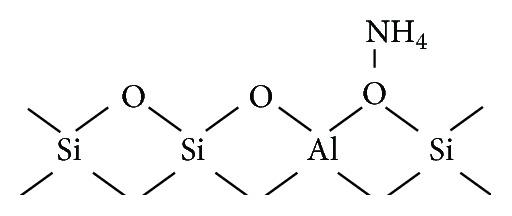
Schematic of NH_4_^+^-type zeolite.

Phosphorylated peptides are very important compounds in biological processes, and the phosphate group exists in several anionic forms in the solution phase. As MALDI MS generally detects singly charged species, MALDI MS measurement of phosphorylated peptides is considered to be difficult. Ammonium salts were used as additives to enhance the peak intensity of phosphorylated peptides by eliminating or lowering the charge effect [14]. In this study, we examined the utility of NH_4_^+^-type zeolite in the MALDI MS measurement of phosphorylated peptides. A schematic of NH_4_^+^-type zeolite is shown in Scheme 1. We expected that the NH_4_^+^-type zeolite would reduce the formation of multiply charged analytes and enhance the peak intensity of the analyte as in the case of ammonium salt. It was found that the NH_4_^+^-type zeolite could reduce matrix-related peaks in the low-molecular-weight region efficiently and enable the detection of phosphopeptides with high intensity.

## 2. Materials and Methods

CHCA, SubP, atropine, aconitine, and a mixture of phosphopeptides were purchased from Sigma Chemical. Zeolites MFI (NH_4_ZSM5), MOR (HM20), and BEA (HB25) were supplied by the Catalysis Society of Japan (CSJ). The SiO_2_/Al_2_O_3_ ratios of the zeolites were 30, 20, and 25, respectively. HZSM5 was obtained by heating NH_4_ZSM5 at 450°C for 3 hours. HM20 was mixed with ammonium acetate solution, and the mixture was stirred for 1 hour at 80°C. After filtration, the residue was washed with distilled water and dried in a vacuum dryer to yield the NH_4_^+^-type zeolite, NH_4_M20.

Four milligrams of each of CHCA and one kind of zeolite was mixed in a mortar for 15 minutes to make zeolite-supported CHCA; this was named “zeolite matrix.” Then, the mixture was dispersed into the mixed solvent of acetonitrile and water (v/v = 1 : 1). The concentration of CHCA in the suspension was adjusted to 4 mg/mL. An analyte was dissolved in the mixture of acetonitrile and water (v/v = 1 : 1). One microliter of each of the zeolite matrix and the analyte solution was pipetted onto a stainless steel plate and mixed several times to induce crystallization. The solvent was allowed to evaporate naturally before MALDI MS analysis.

Mass spectra were obtained with a commercial MALDI MS system (Waters) equipped with a nitrogen laser (337 nm, 10 Hz) in the positive ion reflecting mode. Laser power for excitation was adjusted to 3 *μ*J. Each spectrum was obtained as an accumulation of 400 laser shots.

## 3. Results and Discussion

**Figure 1:**
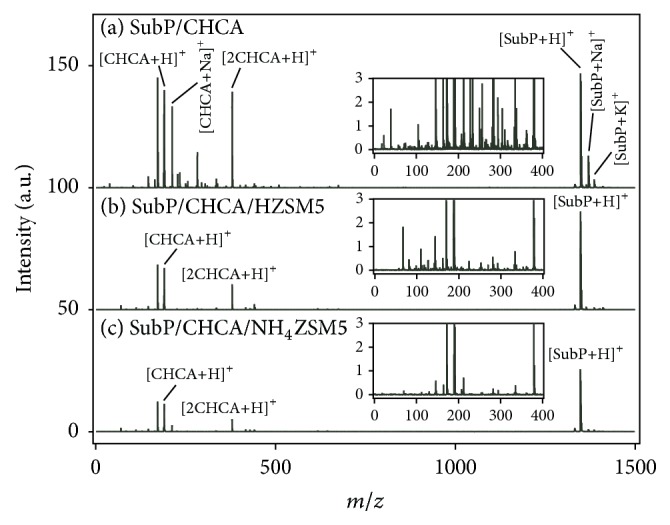
Mass spectra of SubP measured with (a) CHCA only, (b) CHCA/HZSM5, and (c) CHCA/NH_4_ZSM5. Insets are magnifications of the mass region less than 400 Da.

Figure 1(a) shows the mass spectrum of one of the model peptides (Substance P (SubP)) measured with CHCA only. Analyte peaks corresponding to [SubP+H]^+^, [SubP+Na]^+^, and [SubP+K]^+^ were observed at* m/z* = 1348, 1370, and 1386, respectively. In addition, matrix-related peaks, such as [CHCA+H]^+^, [CHCA+Na]^+^, and [2CHCA+H]^+^, were also observed. The peak intensity ratio of [SubP+H]^+^/[CHCA+H]^+^ was 1.17. Insets contain magnifications of the mass spectral region less than 400 Da. A number of peaks with high and low intensities were observed in this mass region. In conventional MALDI MS, the peak intensity of the analyte molecule is usually weak compared with that of the matrix molecule. Therefore, matrix-related ion peaks hinder the identification of analyte peaks if the analyte peaks appear in this mass region. This drawback of conventional MALDI MS could be overcome by using zeolite. Figure 1(b) shows the mass spectrum of SubP measured with a zeolite matrix (CHCA/HZSM5). Here, H^+^-type ZSM5 was used for the measurement. Only the peak of [SubP+H]^+^ was observed, and peaks of [SubP+Na]^+^ and [SubP+K]^+^ were negligible. This advantage was also observed for the matrix molecule; the peak of [CHCA+Na]^+^ disappeared and only the peaks of protonated ions of matrix-related species were observed. The peak intensity ratio of [SubP+H]^+^/[CHCA+H]^+^ (*R*) was 2.35. In addition, we found that many peaks observed in the inset of Figure 1(a) could be suppressed by using zeolite, as shown in the inset of Figure 1(b). Comparing the insets of Figures 1(a) and 1(b), the suppression of matrix-related peaks was quite obvious. Then, NH_4_^+^-type ZSM5 was used to fabricate CHCA/NH_4_ZSM5. In our previous study, we clarified that the Brönsted acidity of the zeolite surface played an important role in the ionization of matrix and analyte molecules [9]. Through NH_4_^+^ termination, however, the number of Brönsted hydroxyl groups would be reduced, and therefore, proton adduction to the matrix and the analyte would be reduced. This was confirmed by measuring SubP with NH_4_^+^-type zeolite. In Figure 1(c), the mass spectrum of SubP measured with CHCA/NH_4_ZSM5 is shown. The peak intensities of [SubP+H]^+^ and [CHCA+H]^+^ became weak (*R* = 2.23). However, NH_4_^+^-type ZSM5 efficiently suppressed the matrix-related peaks; many low-intensity peaks almost disappeared, as shown in the inset of Figure 1(c). Therefore, it was understood that NH_4_^+^-type ZSM5 was more appropriate for the detection of low-molecular-weight analytes than H^+^-type ZSM5, although the peak intensities became weak.

**Figure 2:**
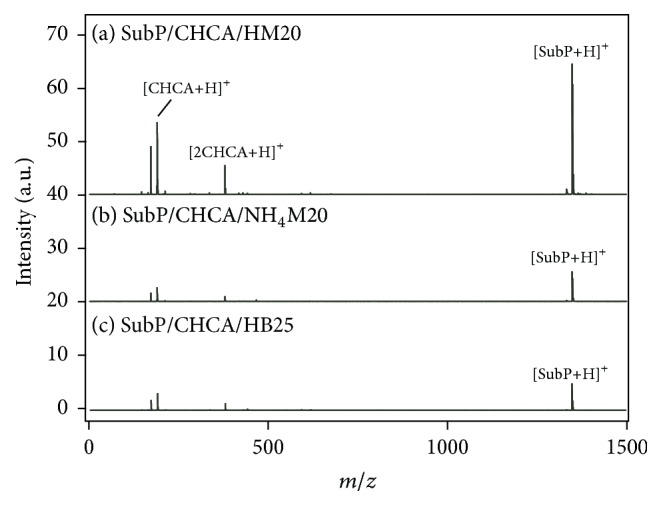
Mass spectra of SubP measured with (a) CHCA/HM20, (b) CHCA/NH_4 _M20, and (c) CHCA/HB25.

Then, we tried to use another type of zeolite (MOR) for the measurement instead of MFI (ZSM5).  Figure 2(a) shows the mass spectrum of SubP measured with CHCA/HM20. The peak of [SubP+H]^+^ was weak compared with Figure 1(b); the peak intensity was ~0.61 times smaller than that with HZSM5 (*R* = 1.81). If we simply assume that the number of hydroxyl groups is related to the number of Si–O–Al bridging sites, the number of hydroxyl groups in HZSM5 would be 0.65 times less than that in HM20; HM20 has many Brönsted OH groups in its structure compared with HZSM5. However, the peak intensity of [SubP+H]^+^ became weak when HM20 was used. When NH_4_^+^-type MOR (NH_4 _M20) was used (Figure 2(b), *R* = 2.12), the peak intensity of [SubP+H]^+^ was approximately 0.22 times smaller than that when NH_4_ZSM5 was used (Figure 1(c)). It is widely known that different kinds of zeolites have different sizes of cavities and channels. For example, MFI has 10- and 8-membered rings whereas MOR has a 12-membered ring. MFI has 0.53 × 0.56 nm^2^ or 0.51 × 0.55 nm^2^ open apertures whereas MOR has 0.65 × 0.70 nm^2^ or 0.26 × 0.57 nm^2^ open apertures [15]. These structural differences are directly related to the catalytic activity of each zeolite. We think that structural differences more strongly affect ionization efficiency than the SiO_2_/Al_2_O_3_ ratio. In fact, when BEA (Beta) zeolite was used for measurement, the peak intensity of [SubP+H]^+^ was much smaller than that when MFI or MOR was used, as shown in Figure 2(c) (*R* = 1.56), although the SiO_2_/Al_2_O_3_ ratio (25) was almost equal to that of MFI or MOR.

**Figure 3:**
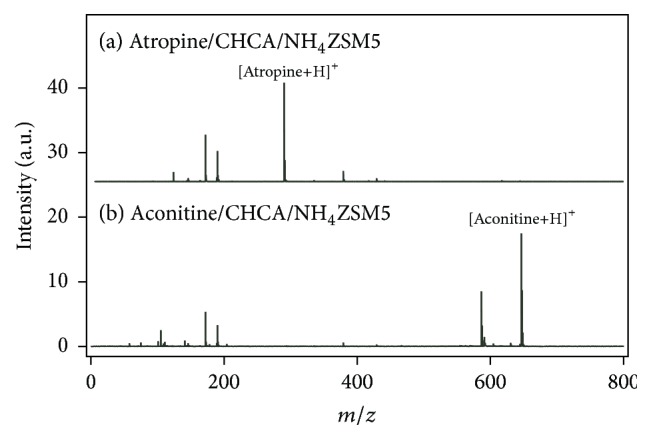
Mass spectra of (a) atropine and (b) aconitine measured with CHCA/NH_4_ZSM5.

Based on the above discussion, it was clarified that NH_4_ZSM5 was useful for the detection of small amounts of low-molecular-weight compounds as the generation of noise peaks in the low mass region could be prevented. Therefore, CHCA/NH_4_ZSM5 was used for the analysis of atropine and aconitine, two toxic alkaloids in plants [16]. Their identification would provide valuable information for the diagnosis of poisoning and drug abuse and shed light on criminal cases. Figures 3(a) and 3(b) show the mass spectra of atropine (2.46 nmol) and aconitine (1.55 nmol) measured with CHCA/NH_4_ZSM5. Intense peaks of protonated atropine and aconitine were observed at* m/z* = 290 and 647, respectively, and peaks of alkali metal ion adducted species were suppressed. Therefore, CHCA/NH_4_ZSM5 could be a beneficial matrix for the detection of low-molecular-weight compounds.

**Figure 4:**
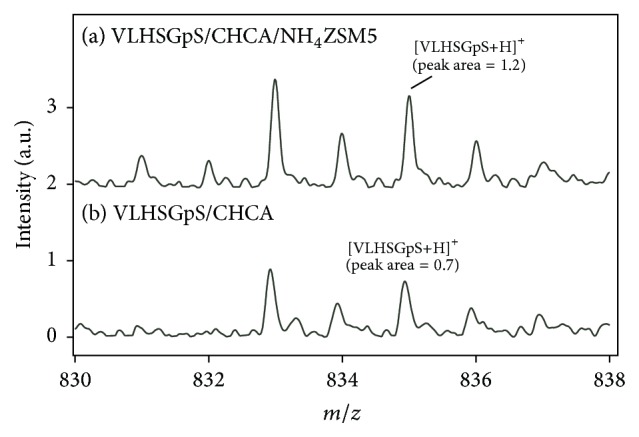
Mass spectra of phosphorylated peptide VLSGpS measured with (a) CHCA/NH_4_ZSM5 and (b) CHCA only.

**Figure 5:**
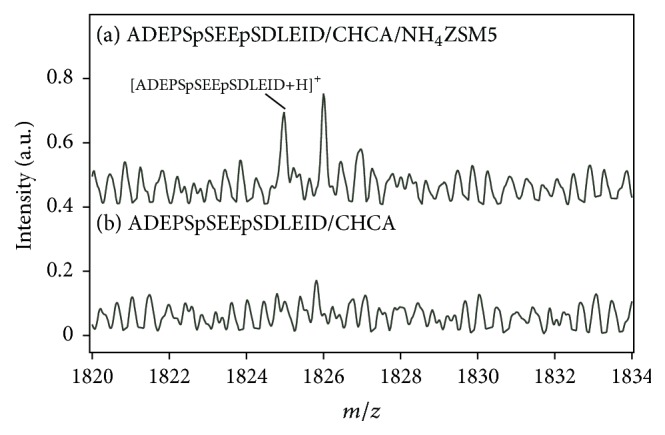
Mass spectra of ADEPSpSEEpSDLEID measured with (a) CHCA/NH_4_ZSM5 and (b) CHCA only.

Finally, CHCA/NH_4_ZSM5 was applied to MALDI MS of phosphorylated peptides. Figures 4 and 5 show the mass spectra of two phosphopeptides in a phosphorylated peptide mixture. The amount of each phosphopeptide in the sample spot was 40 fmol. In the mass spectrum of VLHSGpS shown in Figure 4(a), the peak of protonated VLHSGpS was clearly detected at* m/z* = 835. For comparison, the measurement of VLHSGpS was carried out with CHCA only. The peak intensity of [VLHSGpS+H]^+^ in Figure 4(a) was approximately twofold higher than that in Figure 4(b). Therefore, the ammonium group on the surface was beneficial for lowering the negative charge of the phosphopeptide and enhancing the peak intensity of the protonated ion. Then, MALDI MS was carried out for a large peptide, ADEPSpSEEpSDLEID.  Figure 5(a) shows the mass spectrum of ADEPSpSEEpSDLEID measured with CHCA/NH_4_ZSM5. Although the signal-to-noise ratio was much worse than that shown in Figure 4, the peak of protonated ADEPSpSEEpSDLEID was observed. However, that peak was difficult to detect when only CHCA was used, as shown in Figure 4(b), probably because of the existence of several anionic phosphate groups. Therefore, it was found that CHCA/NH_4_ZSM5 is applicable to the detection of low-molecular-weight compounds having charge deficiency.

## 4. Conclusions

CHCA was adsorbed to zeolite surface, and this complex was applied to the MALDI MS of low-molecular-weight compounds. It was found from the measurement of SubP that the NH_4_^+^-type zeolite could suppress matrix-related peaks more efficiently than the H^+^-type zeolite. CHCA/NH_4_ZSM5 was used for the study of atropine and aconitine, two toxic alkaloids in plants. In addition, CHCA/NH_4_ZSM5 enabled us to detect phosphorylated peptides: peaks of the protonated peptides had higher intensities than the peaks observed with CHCA only.
